# Evaluation of Two Different Strategies for Schistosomiasis Screening in High-Risk Groups in a Non-Endemic Setting

**DOI:** 10.3390/tropicalmed8010044

**Published:** 2023-01-06

**Authors:** Luisa Roade, Elena Sulleiro, Cristina Bocanegra, Fernando Salvador, Begoña Treviño, Francesc Zarzuela, Lidia Goterris, Nuria Serre-Delcor, Inés Oliveira-Souto, Maria Luisa Aznar, Diana Pou, Adrián Sánchez-Montalvà, Pau Bosch-Nicolau, Juan Espinosa-Pereiro, Israel Molina

**Affiliations:** 1 Internal Medicine Department, University Hospital Vall d’Hebron, 08035 Barcelona, Spain; 2 Microbiology Department, University Hospital Vall d’Hebron, PROSICS Barcelona, 08035 Barcelona, Spain; 3 Tropical Medicine and International Health Unit Vall d’Hebron-Drassanes, Infectious Diseases Department, University Hospital Vall d’Hebron, PROSICS Barcelona, 08035 Barcelona, Spain

**Keywords:** schistosomiasis, non-endemic, diagnosis

## Abstract

A consensus on the recommended screening algorithms for schistosomiasis in asymptomatic high-risk subjects in non-endemic areas is lacking. The objective of this study was to evaluate the real-life performance of direct microscopy and ELISA serology for schistosomiasis screening in a high-risk population in a non-endemic setting. A retrospective cohort study was conducted in two out-patient Tropical Medicine units in Barcelona (Spain) from 2014 to 2017. Asymptomatic adults arriving from the Sub-Saharan region were included. Schistosomiasis screening was conducted according to clinical practice following a different strategy in each setting: (A) feces and urine direct examination plus *S. mansoni* serology if non-explained eosinophilia was present and (B) *S. mansoni* serology plus uroparasitological examination as the second step in case of a positive serology. Demographic, clinical and laboratory features were collected. Schistosomiasis cases, clinical management and a 24 month follow-up were recorded for each group. Four-hundred forty individuals were included. The patients were mainly from West African countries. Fifty schistosomiasis cases were detected (11.5% group A vs. 4 % group B, *p* = 0.733). When both microscopic and serological techniques were performed, discordant results were recorded in 18.4% (16/88). Schistosomiasis cases were younger (*p* < 0.001) and presented eosinophilia and elevated IgE (*p* < 0.001) more frequently. Schistosomiasis is a frequent diagnosis among high-risk populations. Serology achieves a similar performance to direct diagnosis for the screening of schistosomiasis in a high-risk population.

## 1. Introduction

Human Schistosomiasis is a neglected tropical disease caused by trematodes of the gender *Schistosoma* spp. Approximately 240 million people worldwide are affected by this condition [1]. Meanwhile, individuals with chronic sequelae after infection are an estimated 440 million [2]. Endemic areas are located in the tropical and subtropical regions of around 70 countries in Asia, America and Africa, although the Sub-Saharan region gathers up to 90% of the cases [1]. Six species of *Schistosoma* sp. affecting humans have been identified whose distribution is related to the fresh-water snail host occurrence [3]. *Schistosoma (S)mansoni, S. japonicum*, *S. mekongi*, *S. intercalatum* and *S. guineensis* invade the mesenteric plexus causing gastrointestinal manifestations, while *S. haematobium* affects the pelvic veins causing genitourinary disease [3]. Acute infection may present with fever and eosinophilia but often goes unnoticed and evolves to chronic forms, which often remain asymptomatic but may lead to severe consequences in the long-term as result of chronic tissue inflammation. 

In low and non-endemic areas, schistosomiasis has gained prominence due to migration streams, increased travel to endemic regions and variations in climate conditions that affect the intermediate host distribution [4,5]. The prevalence in high-risk groups such as migrants from endemic areas could reach up to 15%, and even autochthonous outbreaks in Western countries have been documented [6,7]. The implementation of different screening strategies in non-endemic settings have been proposed for high-risk groups [8].

The direct microscopic observation of eggs in urine or fecal samples by concentration techniques such as Kato-Katz has been considered the reference standard for screening in endemic populations, despite its low sensitivity. Direct observation shows a wide intra- and inter-individual variability due to circadian changes in the egg load and throughout the different phases of the infection, as well as to the observer’s experience [9,10]. Antigenic methods such as circulating antigen detection have been shown to improve the detection of *S. mansoni* infections in these high-risk populations [11]. On the other hand, antibody detection by commercial non-species-specific tests (mainly enzyme-linked immunosorbent assay, ELISA) is widely used in screening protocols for travelers. However, the clinical interpretation of the serological results is often unclear in endemic populations chronically exposed to *Schistosoma* spp. [12]. 

As a result, a consensus on recommended screening algorithms using available techniques is lacking. The aim of our study was to describe two different screening strategies for a high-risk group in a non-endemic area, using direct microscopy observation and serological detection by ELISA respectively, in order to determine accuracy and usefulness in a real-world setting.

## 2. Materials and Methods

### 2.1. Definitions

Both natives and travelers coming from highly endemic areas (mainly Sub-Saharan Africa) were considered as groups at high-risk for the presence of schistosomiasis, with a general expected prevalence of around 15% [6,8], and were therefore candidates for screening.

We use the term *migrant* based on the United Nations Educational, Scientific and Cultural Organization (UNESCO) definition as “any person who lives temporarily or permanently in a country where he or she was not born and has acquired some significant social ties to this country” [13]. Individuals of European origin living in endemic areas were considered *emigrant,* while subjects arriving from endemic areas were considered *immigrants.* The term *recently arrived migrant* was applied to individuals who had arrived in Europe up to 6 months before inclusion. A subgroup of *visiting friends and relatives* (VFR) was categorized according to the Centers for Disease Control and Prevention (CDC) definition as those migrants who had ever returned to their home country since their arrival to Europe [14].

### 2.2. Study Design

A retrospective longitudinal cohort study was performed in order to compare two different screening strategies for schistosomiasis used in the clinical practice.

### 2.3. Settings

The study was conducted in two out-patient units specialized in tropical medicine, belonging to the International Health Program of Catalan Institute of Health (PROSICS) in Barcelona, Spain. Both units provide free medical care to self-referred individuals and referred cases from general practitioners and other medical specialists, public health services and non-governmental organizations (NGOs). Each clinic performs a different schistosomiasis screening strategy: (A) hemogram and feces and urine concentration techniques for direct examination, followed by *S. mansoni* serology if non-explained eosinophilia was present, and (B) *S. mansoni* serology and uroparasitological direct examination at a later time in case of a positive serology. Coproparasitological analysis was also performed in group B patients as part of general parasitosis screening, but not specifically for *Schistosoma* screening. All samples were analyzed at the Parasitology Unit of Vall d’Hebron Microbiology Department.

### 2.4. Study Population and Data Collection

Asymptomatic adults arriving from the Sub-Saharan African (SSA) region from January 2014 to December 2017 were included. Subjects with a previous history of either eosinophilia or high levels of immunoglobulin E (IgE), schistosomiasis or macrohematuria were excluded. Demographic, clinical and laboratory data at baseline were collected in all subjects. 

Laboratory tests included hemogram [with absolute eosinophil count (AEC)] and biochemical panel. Mild eosinophilia was defined as AEC between 500 and 1000 cells/µL, moderate eosinophilia as AEC between 1000 and 3000 cells/µL and severe eosinophilia as AEC ≥ 3000 cells/µL. The upper normality limit for relative eosinophils count was settled in 4.5%, according to the recommendations of the expert group of the Spanish Society of Tropical Medicine and International Health (SEMTSI) for the diagnosis and treatment of imported eosinophilia [15]. Total serum IgE levels were also recorded when available and considered normal below 500 KU/L, following the local reference threshold. Routine migrant screening, including viral hepatitis serology (hepatitis B surface antigen and anti-hepatitis C antibodies), human immunodeficiency virus (HIV) antibodies and antigen-p24 detection and syphilis serology (TPHA and RPR), were performed in all subjects. Asymptomatic individuals aged below 35 years were also tested for tuberculosis infection by either tuberculin skin test or tuberculosis blood test (Quantiferon-TB Gold^®^). Other latent infections, such as malaria and filariasis, were screened based on the patient’s individual risk assessment. Microscopic examination of stool and urine was performed through concentration techniques. Stool examination was carried out using the Ritchie’s formalin-ether technique, while urine samples were processed by centrifugation. The identification of the different *Schistosoma* species was established according to the morphologic characteristics of the eggs. An enzyme-linked immunosorbent assay (ELISA) for IgG against *S. mansoni* (Novagnost *S. mansoni* IgG; Siemens Diagnostics, Marburg, Germany) was performed following manufacturer instructions. The results were expressed by index and considered positive when the index was ≥1.1, negative when the index was ≤0.9 and grey zone in values ranging from 0.9 to 1.1.

**Figure 1:**
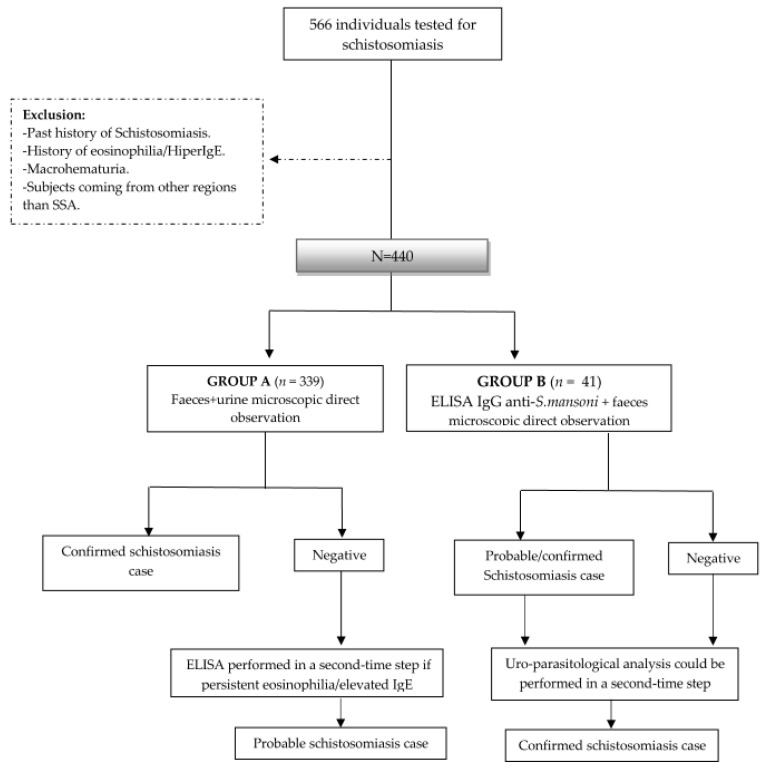
Study design and flowchart. *ELISA* enzyme-linked immunosorbent assay; *IgE* immunoglobulin E; *IgG immunoglobulin G; S. mansoni* Schistosoma mansoni; *SSA* Sub-Saharan Africa.

Complementary examinations [abdominal ultrasound (US) and X-ray] were performed at the discretion of the physician in charge. Treatment regimens and follow-up at month 6, 12 and 24 after therapy were also recorded. Figure 1 shows a flowchart of the study design.

### 2.5. Case Definition

*Confirmed cases* were established after direct observation of *Schistosoma* eggs in either feces or urine samples. They were classified as either intestinal or genitourinary disease according to the identified *Schistosoma* species [16]. A *probable* schistosomiasis case was defined by a positive serology, which was considered clinically relevant (this is followed by a subsequent treatment prescription) by the responsible physician, without parasitological confirmation. The disease location was considered undetermined due to the inability to identify the parasite species in probable cases. Cases considered false positive according to the medical records were also recorded. 

### 2.6. Statistical Analysis

Univariate analysis of the dataset included measures of distribution, central tendency (median or mean depending on distribution), and dispersion (standard deviation or interquartile range [IQR]). Normally distributed quantitative variables were compared with the Student *t*-test. Non-normally distributed quantitative variables were analyzed with the Mann-Whitney U test. Categorical variables were described in frequency and percentage. The bivariate analysis was carried out using the χ2 test or the Fisher’s exact test for frequencies below 5%. Hypothesis testing was conducted with a 5% alpha risk and 95% confidence intervals (CI), and considered statistically significant if the two-tailed *p*-value was below 0.05. Statistical analysis was conducted using IBM SPSS, version 26.0 for Windows (SPSS Inc., Armonk, NY, USA).

### 2.7. Ethics Statement

Ethical approval was obtained from the Ethical Committee of the University Hospital Vall d’Hebron PR(AG)112/2016. All data of the subjects participating in the study was anonymized. The need for informed consent was waived due to the study’s design. 

## 3. Results

### 3.1. Baseline Features

A total of 566 patients were tested for schistosomiasis in both settings during the study period. Four-hundred and forty patients met the inclusion criteria and were included in the analysis; 399 (90.7%) were screened by urine and feces examination (group A), while 41 (9.3%) were screened by serology (group B). The flowchart is summarized in Figure 1. 

Table 1 summarizes the baseline characteristics of the overall cohort and by screening strategy. Most subjects were male (56.4% in group A vs. 61.0% in group B) with an overall median age of 36 years (36 vs. 32 years, *p* = 0.06). Most of the cohort arrived from Western Africa; predominantly from Equatorial Guinea (190 subjects, 43.2% of the overall cohort). 

**Figure 2:**
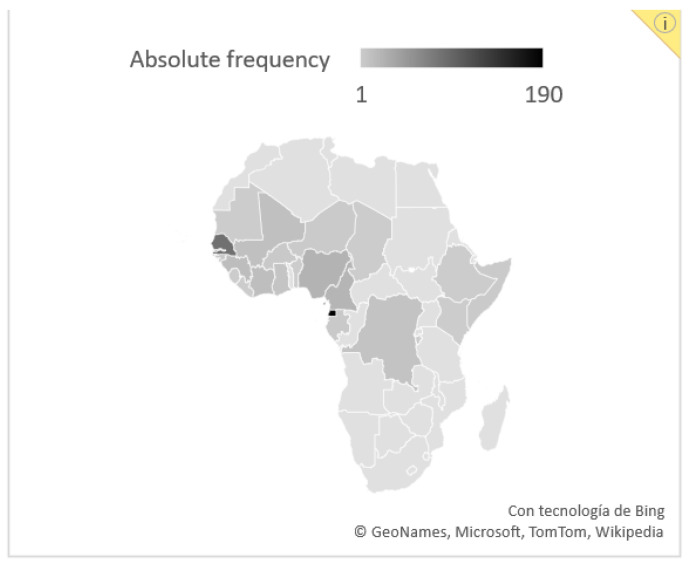
Country of origin of individuals in overall cohort (*n* = 440) by absolute frequency.

The country of origin of the individuals from the general cohort are represented in Figure 2. 

A vast majority of the subjects (372, 84.5%) did not have a significant past medical history. Fifteen subjects (3.4%) presented known HIV infection, with a significantly higher proportion in group B (2.8 vs. 9.8%, *p* = 0.019). Sixteen subjects (3.6%) had a previous history of chronic viral hepatitis (9 cases of hepatitis B, 6 cases of hepatitis C and 1 coinfection). Notably, known history of cardiovascular risk factors (i.e., dyslipidemia, arterial hypertension and diabetes mellitus) prevailed over infectious diseases in the general cohort (43 subjects [9.8%] vs. 31 [7.0%]). 

### 3.2. Schistosomiasis Screening Outcomes

Fifty subjects (11.4%) were diagnosed with a *Schistosoma* infection during the study period. The general features of the schistosomiasis cases and the comparison to subjects without schistosomiasis are presented in Table 2. Absolute and relative eosinophilia were more frequent in the schistosomiasis group (50% vs. 12.6% in absolute count, 82% vs. 31.3% in relative count, both *p* < 0.001). 

**Figure 3:**
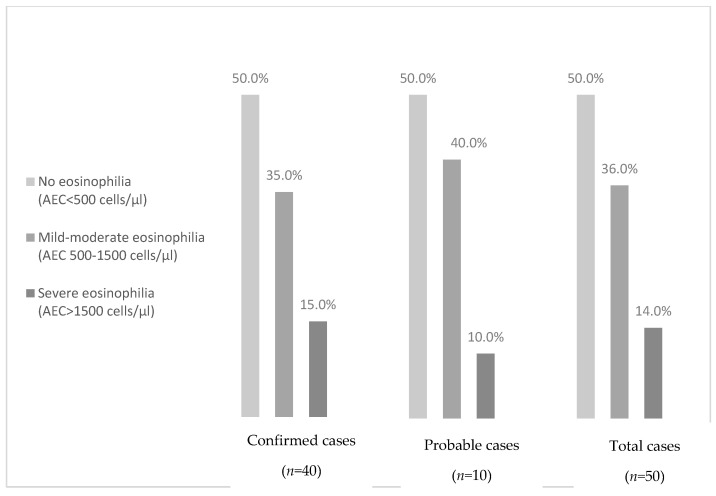
Grades of eosinophilia (by relative frequency) in schistosomiasis cases.

Grades of eosinophilia in schistosomiasis cases are represented in Figure 3. No significant differences were found in the presence of eosinophilia between subjects with confirmed probable schistosomiasis. 

The proportion of schistosomiasis cases did not differ significantly r between both strategies (46 cases [11.5%] in group A vs. 4 cases [9.8%], *p* = 0.733). According to the screening strategy:

#### 3.2.1. Group A

In group A, based on copro and uroparasitological direct examination, 46 cases (11.5%) of schistosomiasis out of 399 screened subjects were detected. Thirty-seven (9.3%) cases were diagnosed by urine and/or feces examination; the vast majority were stool samples (30 cases; 26 subjects presented *S. intercalatum* eggs, four cases with *S. mansoni* eggs). Uro-parasitological samples yielded positive results in six subjects, in which *S. haematobium* eggs were observed. One case with positive results in both stool (*S. intercalatum*) and urine (*S. haematobium*) examination was detected. Serology was performed in a second-step diagnosis as part of an eosinophilia study in two subjects with confirmed gastrointestinal schistosomiasis by *S. intercalatum* and *S. mansoni*, respectively, and results were negative in both cases. Serology was performed for a second time in 45 subjects with previous negative results in urine and stool microscopic observation as part of the investigations for eosinophilia and/or hyper IgE found in baseline blood test. Eleven subjects tested positive, although a probable schistosomiasis was assumed in nine cases (81.8% of the cases with a positive ELISA result). The remaining two cases presented a positive serology for Strongyloides stercolaris (high titles) and were diagnosed as such, considering *Schistosoma* spp. serology positivity due to probable cross-reaction. 

#### 3.2.2. Group B

Screening was conducted through baseline *Schistosoma* ELISA serology in 41 cases. The results were positive in six individuals, two of which were not interpreted as clinically relevant according to medical records. No cases were detected by stool analysis. Among the four probable cases, three subjects underwent uroparasitological examination. Genitourinary schistosomiasis was confirmed in all three cases with the observation of *S. haematobium* eggs in urine specimen. 

Table 3 shows the correlation between serology and copro/uroparasitological studies when both the direct examination technique and serology were performed. Both tests were performed in 88 subjects. Sixteen (18.4%) of them showed discordance between the different techniques (either positive serology with negative uroparasitological examination or positive microscopic observation with negative serology). Out of the seventeen subjects with a positive serology, no *Schistosoma* spp. eggs were found in fourteen (82.4%); among seventy patients with negative serology, eggs were found in two (2.9%). 

### 3.3. Schistosomiasis Treatment, Complementary Examinations and Follow-Up

Among 50 subjects with schistosomiasis diagnosis, 46 received antiparasitic treatment with praziquantel. Four subjects (8.0%) were lost in the follow-up before receiving treatment. Treatment regimens were non-systematically collected in medical records. The most commonly therapeutic scheme was 40 mg/kg/day, administered twice a day on two consecutive days (34 cases, 68%); however, other therapeutic regimens, such as 40 mg in a single dose, or repeated doses 15 days later, were also administered. Concerning complementary examinations, abdominal US was performed in 12 subjects (24%). Pathological findings were present in four of them (33.3%), consisting of bladder wall irregularities and thickening in all cases.

Schistosoma serology after a 6-month follow-up was performed in seven subjects who had previously tested positive. Serological test persisted as positive in six of them, and titles did not present a significant decrease in any of them. Serology was repeated after 12 months in two subjects, showing a persistent positive result in both. Re-treatment was administered in one case due to persistent eosinophilia.

### 3.4. General Screening Results

**Figure 4:**
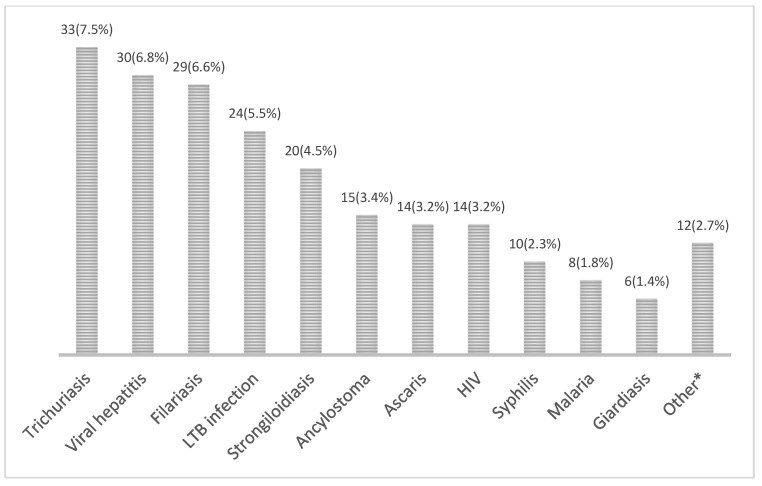
Absolute and relative frequency of newly diagnosed infections in the general cohort (*n* = 440). *HIV* human immunodeficiency virus; *LTB* latent tuberculosis. * The category “other” included cases of amebiasis, gonorrhea and *Mansonella*, *Toxocara canis*, *Taenia solium* and *Dicrocoelium dendriticum* infections.

From the overall cohort, 188 subjects (42.7%) were diagnosed with 215 other infections during systematic screening; 45 individuals (23.9%) showed concomitant coinfections. Among *Schistosoma*-infected subjects, 26 (52.0%) were diagnosed with other infectious diseases. The most frequent diagnosed infections in the overall cohort are summarized in Figure 4. 

Coinfections were more frequent in subjects with baseline eosinophilia (71.6% vs. 36.9%, *p* < 0.001). Among the twenty-five schistosomiasis cases with baseline absolute eosinophilia, 14 (56%) were diagnosed with any other infections. Considering both previously known and newly diagnosed cases, chronic conditions such as chronic viral hepatitis and HIV reached 10.5% and 6.6% prevalence in the overall cohort, respectively. 

## 4. Discussion

Our study analyzed two schistosomiasis screening strategies used in the clinical practice of an International Health Institution in Barcelona, Spain, from 2014 to 2017. All diagnosed cases correspond to immigrants, mostly from Equatorial Guinea. This country is probably over-represented due to historical and cultural ties with Spain and the high degree of awareness of the importance of screening in this group. Similar demographic characteristics have been reported in cohorts within the Spanish territory [17,18]. 

The rationale for screening schistosomiasis in a population from endemic areas is that the disease is highly prevalent, and treatment with praziquantel is safe and effective [18,19]. Studies have reported a prevalence of schistosomiasis ranging from 20 to 40% in endemic populations [19,20]. In migrants, the prevalence ranged from 1% to 18% depending on the screening strategy (parasitological vs. serological study), according to a recent meta-analysis [21,22,23]. In our study, the overall detected prevalence including both screening strategies was 11.5%.

It should be noted that the accuracy of the parasitological study depends largely on the experience of the observer, and therefore may vary significantly between laboratories. Moreover, in contexts with a low egg burden, egg excretion may present great variations throughout the day; even in the same sample, the eggs may be unevenly distributed [24,25]. The use of automatized techniques would allow screening to be expanded and performed in primary care units, and not necessarily in specialized centers.

In this situation, the sensitivity of the technique should be prioritized over specificity, although performing two tests in parallel could be used to increase the accuracy of detection and inform schistosome species [8]. In our study, the percentage of diagnosed cases were similar in both described strategies. A possible explanation could be the high specialization of the reference laboratory, which optimizes the sensitivity of direct observation techniques. This condition might not be generalized to other contexts with less specialized laboratory personnel. Nevertheless, in the case of intestinal schistosomiasis, other more sensitive techniques, such as the saline gradient or the helminthex method, could improve the sensitivity of direct diagnosis [26]. 

In this study, the serology used was an ELISA based on crude antigens of *S. mansoni*. Several studies show that this technique is more sensitive than the direct visualization of eggs, especially in adults with light infections from non-endemic areas [27,28]. Nevertheless, the sensitivity of the serological tests for other *Schistosoma* species could be compromised. Thus, sensitivity varies between 50 and 90% for *S. mansoni* and 20 and 70% for *S. haematobium* [29].

The prevalence of imported eosinophilia among travelers and immigrants is reported between 8% and 28.5%, consistently with the reported prevalence of 16.8% in our cohort. Etiological diagnosis is often troublesome. Depending on the depth of the study and the population analyzed, a parasitic cause is identified in 17% to 75.9% of individuals. Among the difficulties encountered to compare studies are the heterogeneity of the studied populations, the study designs and the different diagnostic protocols [15]. In schistosomiasis, eosinophilia is not a consistent finding [30,31], especially in adult migrants in whom infection has probably happened in childhood and presented a low parasite load. In our study, 50% had eosinophilia. However, this finding could be influenced by the usual presence of coinfections, such as strongyloidiasis, filariasis and soil-transmitted infections. Consistent with other studies [18,32], IgE elevation was more prevalent than eosinophilia (68%). However, one third of the tschistosomiasis cases presented no abnormal IgE levels. Therefore, screening of the disease should be based primarily on origin in the case of migrants, and on risk activities in the case of travelers, rather than on the presence of these parameters [21].

Once screening has been performed, the exact procedure to follow once individuals are considered positive is not clear. There are few guidelines on the management of these patients, either symptomatic or asymptomatic. Some guidelines recommend performing a parasitological examination in the case of a positive serology, and performing an ultrasound only if eggs are found [33]. Although this is essential to differentiate the species, the low sensitivity of the parasitological test and the presence of lesions in patients without egg detection suggest that an ultrasound should be performed in all patients, regardless of the parasitological result and the presence of symptoms [34,35]. In our study, in all patients diagnosed either by parasitological study or by serology, treatment was considered. However, the percentage of ultrasounds performed was very low, reflecting the difficulties on an appropriate management in many contexts. One third of the performed ultrasounds showed pathological findings related to the infection.

One limitation of schistosomiasis serology is that its use to differentiate between current and past infection is very limited, since antibody titers vary significantly after administering an adequate treatment [36]. In our study, only 2.5% of patients underwent control serology, of which 73% resulted positive. Follow-up of migrant subjects is difficult, especially regarding screening of asymptomatic populations, due to socioeconomic and linguistic barriers, high mobility and lack of disease perception [37]. Mechanisms need to be in place to ensure non-discrimination in access to health care, such as availability of cultural mediators, improvement of health literacy through targeted health promotion interventions, supranational communication system and effective asylum policies, among others [38,39].

Due to the limitations of both techniques currently used, new tests with higher sensitivity and specificity that are capable of differentiating current from past infections should be developed. Meanwhile, the combination of several serological techniques, the western blot technique, or the immuno-chromatographic test (ICT) have shown a higher sensitivity [29].

A recent evaluation of several diagnostic tools found that a rapid commercial serological ICT test with a 96% sensitivity would have the potential for being used as a single screening test for migrants from Sub-Saharan Africa [40]. Molecular techniques (serum or excreta PCR) also have a high performance, improving the sensitivity of serology and allowing confirmation of the *Schistosoma* species involved. Furthermore, they can be useful for outcome monitoring [41]. More efforts should be made to make these tests widely available for their use in daily practice. Lateral flow (LF) essays for the detection of worm-related circulating antigens have also been developed in urine and blood-related products [42,43]. LF-circulating anodic antigen (CAA) in serum has shown to be useful in order to monitor treatment response, although wider evidence is needed for non-endemic areas [42,43].

Regarding schistosoma treatment, wide heterogenicity has been observed in prescribed antiparasitic regimens [44,45]. In our cohort, the most commonly prescribed therapeutic scheme (40 mg/kg/day, twice a day during two consecutive days) overcame the dosages recommended by WHO (40 mg/kg/day) and CDC (40–60 mg/kg/day one single day). However, these are recommendations for disease control in endemic areas; an increase in dosages and duration of antiparasitic treatment were common in a recent review of cases from non-endemic areas, emphasizing the need for randomized clinical trials in this setting [44]. Regarding general screening, an important proportion of the cohort was diagnosed with some infectious diseases, which encourages the maintenance of screening programs with the active participation of actors such as NGOs, community organizations and other health care departments. The lower rate of infections in our study compared to similar publications could be probably explained due to the exclusion of individuals with symptoms or known eosinophilia/elevated IgE [17,18]. Concerning general migrant health status, it is worthy to report the high prevalence of cardiovascular conditions, even among such a young population as that included in this cohort [46]. Specific, culturally oriented strategies and resources should be available to ensure the detection and management of non-communicable diseases in these vulnerable populations [47].

This study presents important limitations, mainly due to its retrospective design. Information bias due to incomplete data in medical records could have influenced the results concerning the serological interpretation. As foresaid, selection bias could not be completely avoided, despite the tight inclusion criteria. Also, the sample size of both strategies considerably differs, making difficult a significant comparison between groups. In the same line, the sample of schistosomiasis cases is too limited to draw robust conclusions. The high rate of loss at follow-up, related to the precarious life conditions, is the cause of a substantial amount of data losses, especially concerning follow-up information. Finally, it is worthy remarking that inclusion was limited to asymptomatic subjects coming from SSA; thus, the results might not be generalized to other populations. Despite these remarkable limitations, we believe that this study provides valuable data on the screening of Sub-Saharan migrants in the clinical practice.

## 5. Conclusions

Serology achieves a similar performance to direct diagnosis for the screening of schistosomiasis in a high-risk population. Due to the standardization of the serology technique and its availability at all levels of care, facilitating access to all patients, this strategy is recommended. Screening should be based primarily on origin rather than on the presence of eosinophilia and/or elevated IgE levels, due the inconsistence of these parameters.

## Figures and Tables

**Table 1:** Baseline characteristics in the overall cohort and by screening strategy.

	Overall (*n* = 440)	Group A (*n* = 399)	Group B (*n* = 41)	*p*-Value
Male *	250 (56.8%)	225 (56.4%)	25 (61.0%)	0.32
Age (years) **	36.0 [20]	36.0 [20]	32.0 [18]	0.06
Country of origin * − **Equatorial Guinea**				
	190 (43.2%)	178 (44.6%)	12 (37.1%)	0.06
Past medical history * − **HIV** − **Chronic viral hepatitis** − **Cardiovascular risk-factors**	15 (3.4%) 16 (3.6%) 43 (9.8%)	11 (2.8%) 14 (3.5%) 41 (10.3%)	4 (9.8%) 2 (4.9%) 2 (4.9%)	0.02 0.66 0.27
Type of migrant * − **Emigrant** − **Immigrant** **VFR** **Newly arrived**	13 (3.0%) 427 (97.0%) 145 (33.0%) 157 (35.7%)	7 (1.8%) 392 (98.2%) 136 (34.1%) 139 (34.8%)	6 (14.6%) 35 (85.4%) 9 (22.0%) 18 (43.9%)	<0.001 - 0.03 0.26
Peripheric eosinophilia *	82 (18.6%)	72 (18.1%)	10 (24.4%)	0.32
Elevated IgE *	242 (55.0%)	239 (61.3%)	3 (100%)	<0.001

* frequency (%); ** median [IQR]. *HIV* human immunodeficiency virus; IgE total immunoglobulin E; *VFR* visiting friends and relatives. Peripheric eosinophilia was determined as absolute eosinophile count above 500 cells/µL; Elevated IgE was defined as total IgE levels above 117 KU/L.

**Table 2:** Schistosomiasis cases diagnosed by screening strategy.

Schistosomiasis Cases *	Group A (*n* = 399)	Group B (*n* = 41)
**Confirmed cases** − ***S. haematobium*** − ***S. mansoni*** − ***S. intercalatum***	**37 (9.3%)** 26 (6.5%) 4 (1.0%) 6 (1.5%)	**3 (7.3%)** 3 (7.3%) - -
**Probable cases**	9 (2.0%)	1 (2.4%)
**Total**	46 (11%)	4 (9.8%)

* *n* (%). Probable cases were defined by a positive serology considered as clinically relevant by the physician in charge. Confirmed cases were established after direct observation of *Schistosoma* spp. eggs in excreta.

**Table 3:** Correlation between serology and copro-uroparasitological examination.

		Copro/Uroparasitological Sample Examination				
		*S. mansoni*	*S. intercalatum*	*S. haematobium*	No eggs	Total
***S. mansoni.* serology (ELISA IgG)**	Positive	0 (0%)	0 (0%)	3 (17.6%)	14 (82.4%)	17
	Negative	1 (1.4%)	1 (1.4%)	0 (0%)	69 (97.2%)	71

## Data Availability

Not applicable.
